# Distributional Patterns of *Pseudacteon* Associated with the *Solenopsis saevissima* Complex in South America

**DOI:** 10.1673/031.009.6001

**Published:** 2009-08-20

**Authors:** Richard J. W. Patrock, Sanford D. Porter, Lawrence E. Gilbert, Patricia J. Folgarait

**Affiliations:** ^1^ Centro de Estudios e Investigaciones, Universidad Nacional de Quilmes, B1876BXD Bernai, Buenos Aires, Argentina; ^2^ Section of Integrative Biology and Brackenridge Field Laboratory, University of Texas, Austin, Texas 78712 USA; ^3^ USDA-ARS, Center for Medical, Agricultural and Veterinary Entomology, 1600 SW 23rd Drive, Gainesville, FL 32604, USA

**Keywords:** classical biological control, imported fire ants, parasitoid, Phoridae, Formicidae, geographical ranges, host use patterns

## Abstract

Classical biological control efforts against imported fire ants have largely involved the use of *Pseudacteon* parasitoids. To facilitate further exploration for species and population biotypes a database of collection records for *Pseudacteon* species was organized, including those from the literature and other sources. These data were then used to map the geographical ranges of species associated with the imported fire ants in their native range in South America. In addition, we found geographical range metrics for all species in the genus and related these metrics to latitude and host use. Approximately equal numbers of *Pseudacteon* species were found in temperate and tropical regions, though the majority of taxa found only in temperate areas were found in the Northern Hemisphere. No significant differences in sizes of geographical ranges were found between *Pseudacteon* associated with the different host complexes of fire ants despite the much larger and systemic collection effort associated with the *S. saevissima* host group. The geographical range of the flies was loosely associated with both the number of hosts and the geographical range of their hosts. *Pseudacteon* with the most extensive ranges had either multiple hosts or hosts with broad distributions. Mean species richnesses of *Pseudacteon* in locality species assemblages associated with *S. saevissima* complex ants was 2.8 species, but intensively sampled locations were usually much higher. Possible factors are discussed related to variation in the size of geographical range, and areas in southern South America are outlined that are likely to have been under-explored for *Pseudacteon* associated with imported fire ants.

## Introduction

Knowledge of species distributions is a fundamental component of all areas of applied ecology. This information is particularly crucial in applied ecology. From conservation and comparative ecology to assessments of human effects on the environment, the distribution of species is an important variable used to evaluate effects and establish comparative standards. Foreign exploration for natural enemies of introduced pests, for instance, is the primary step in classical biological control operations and requires at least a generalized knowledge of where to begin (Bartlett and van den Bosch 1964). It is more likely that researchers will meet their objectives of finding population sources offering sufficient numbers and the appropriate qualities of natural enemies if they have a better understanding of the pest and the distribution of its natural enemies. Typically, one of the outcomes of this type of work is detailed distributional information that will prove useful for understanding the ecological relationships between the target hosts and the natural enemies.

Currently, there is an international biological control program aimed at management of the imported fire ants, *Solenopsis invicta* Buren and *S. richteri* Santschi. While efforts are being made to incorporate a variety of pathogens and others, the focus of much of the work has been on the introduction of phorid parasitoids in the genus, *Pseudacteon* (Porter et al. 1995; Gilbert and Patrock 2002; Williams et al. 2003). Phorids were first advocated for use against imported fire ants by Williams et al. (1973) and there have been a series of explorations for these flies over much of the native range of these ants in South America (Williams et al. 1974; Folgarait et al. 2005; Calcaterra et al. 2005; Calcaterra et al. 2007; Folgarait et al. 2007a, b).

Host-specificity of many of the species tested in the laboratory (Estrada et al. 2006; Porter and Gilbert 2004 and references therein) indicates that these species have very narrowly defined taxonomic host use that may be further specialized at the population level. An important taxonomic facet of this host specificity is that *Pseudacteon* associated with the genus *Solenopsis* appear to belong to two mutually exclusive groups, one hosted by species in the *S. geminata* complex and the other hosted by species in the *S. saevissima* complex (Trager 1991). This host division has biogeographic components in that *Pseudacteon* species associated with either host group have not switched to the other group despite host and parasitoid overlap in some areas of their distributions (Fowler et al. 1995; Porter et al. 1995).

There have been a number of successful releases of *Pseudacteon*, including *P. tricuspis* Borgmeier, *P. curvatus* Borgmeier and *P. litoralis* Borgmeier in the United States (Callcott and Weeks 2007). The overall outcome of these trials has indicated that additional species or different climate ecotype populations are required for fire ant management (Gilbert and Patrock 2002). To facilitate further exploration for suitable populations of these flies, collection data were organized from a number of sources to determine known distributions of the *Pseudacteon* associated with the *S. saevissima* complex in order to determine priority areas for collecting. While the focus was on this group of phorids, a database was also developed for other *Pseudacteon.* This added information was included in the analyses to provide a spatial and sampling context for the subset of the phorid genus.

## Materials and Methods

Distributional data were collected from the literature, from specimens in our own collections and from major museum holdings of *Pseudacteon*, including the Museu de Zoologia, Universidade de São Paulo, Brazil (MZSP), Natural History Museum of Los Angeles Co. (LACM), Museo Argentino de Ciencia Naturales, Buenos Aires (MACN), Instituto Miguel Lillo, Tucumán, (IML) and the Texas Memorial Museum (TMM). A few records were found by combing through on-line search engines for 32 entomological museums. Where geographical reference points were not given, GPS coordinates were approximated using a variety of sources. Distributional maps were then drawn using Arc View 9.1 (Gorr and Kurland 2005). Two estimates of geographical range were calculated for all of the *Pseudacteon* species, including Latitudinal Range and Range Distance (greatest distance between any two collecting spots of the range). Range Distances for each species were determined using the Latitude/Longitude Distance Calculator found at http://www.nhc.noaa.gov/gccalc.shtml.

Several questions are addressed pertaining to variation in *Pseudacteon* geographical ranges using correlation analyses and MANOVA within R (R Development Core Team 2006). The data that were collected included geographical information as well as dates and years of collection, numbers of collecting records, numbers of individuals collected and host information. For the correlation analyses, it was hypothesized that geographical ranges would be related positively to numbers of hosts/taxa, as well as collection intensity, the latter which was represented by 1) the number of years that a taxon had been collected, 2) number of months of the year over which a taxon was found and 3) the number of localities where each taxon had been found. The questions addressed in MANOVA related to whether geographical ranges differed according to host complex and/or to the geographical region examined. Finally, species richness patterns, or assemblages of *Pseudacteon* at different localities were described. In some cases, the data were from a set of records in the same locality but with different collectors and years. To account for these cases, the locality assemblage was defined broadly as the taxa within a 0.1° latitude-longitude area, which was the resolution for many of the records.

**Figure 1.**
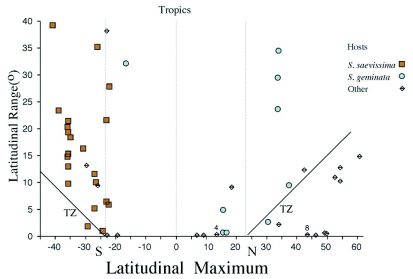
Bivariate plot of *Pseudacteon* species latitudinal maxima and latitudinal ranges. Latitudinal maxima are the maximum absolute latitudinal values for each species. These values were then reset to their original North-South polarity. Values between the lines extending from -23.5 and 23.5 are for species known only from the tropics, while those above the lines marked TZ (Temperate Zone) represent those species whose ranges extends into the tropics. Below the TZ lines, the species are exclusively temperate. Ranges for species in the northern and southern hemisphere extend south and north from their maxima, respectively.

Each of the variables was transformed to best-fit assumptions of normality. Distance was log-transformed, number of years, months/year and locality numbers were square-root transformed. Bonferroni's method was used to adjust P-values to accommodate multiple tests using the same data.

## Results

### General patterns

Records were found for 57 species of *Pseudacteon* including five species that are not currently assigned a taxonomic binomial (Brown and Feener 1998; Calcaterra et al. 2005; Philpott 2005; Wild 2007; Kronfurst et al. *in press*). Additional collection records without names or host records indicate this species count may be a substantial underestimate, particularly in the Old World. *Pseudacteon* species have been collected in all the major biogeographical regions with records known for 61 nations. Of those species named, 41 are New World (Nearctic (11), Neotropical (35)); 12 species are Palearctic and 3 species have been found in the Oriental Region. Twenty-one and nine species of New World *Pseudacteon* are associated with hosts in the *S. saevissima* and *S. geminata* complexes, respectively (Appendices 1–3).

*Pseudacteon* species have been found as far north as 61.8° (*P. fennicus*) and as far south as 41.°S (*P. obtusus*, Appendix 1). The mean latitudinal and longitudinal ranges for the genus were 15.3 ± 10.8° and 17.6 ± 12.0° (N = 40), respectively. The mean Range Distance for the genus was approximately 2081 ± 1449 km (Appendix 1). New World species had significantly longer Range Distances than Old World species (Mann-Whitney U, Z = -3.1, P = 0.0018).

Latitudinal ranges are plotted against maximum latitudinal occurrence for each *Pseudacteon* species in Figure 1. The distribution of taxa was spread evenly across latitudes with 16 tropical species, 18 temperate species, and 20 species having ranges that extended across both temperate and tropical zones (Figure 1). The majority of taxa with only temperate distributions were found in the northern hemisphere (Figure 1, Appendix 1). With respect to those species associated with the *S. saevissima* complex only, *P. bulbosus* was not been found within the tropics, and four species, *P. affinis, P. dentiger, P. fowleri* and *P. lenkoi* are known only from the tropics (Figure 1, Appendix 2).

### Host association patterns

Geographical breadth of *Pseudacteon*, as measured by Range Distances, was significantly related to each of the measures of collecting intensity, including Years since First Collection (R = 0.41, P = 0.009), Number of Localities (R = 0.36, P = 0.03), and Number of months/year collected (R = 0.45, P = 0.003). The partial correlation between Range Distances and Number of Associated Hosts (that is, after adjusting for the measures of Collection Intensity) was not significant, however, (All species, R = 0.08, P > 1.0, *S. saevissima* complex flies, R = -0.07, P> 1.0).

Geographical ranges for species hosted by the two *Solenopsis* species complexes were not significantly different as estimated by Latitudinal Range (MANOVA F_1,26_ = 0.97, P = 0.34) or Range Distance (MANOVA F_1,26_ = 0.25, P = 0.62) although on average, species hosted by the *S. geminata* complex had both wider mean Latitudinal Ranges (19.6 ± 13.5 vs. 16.2 ± 10.1°) and Range Distances (3232 ± 2115 vs. 2047 ± 1048 km) than those for the *S. saevissima* group.

Collection intensity, as measured by locality records, that is the number of 0.1° latitude-longitude areas (Appendix 1) was significantly greater for the *S. saevissima* complex *Pseudacteon* than for *Pseudacteon* in North America or the Old World (Sqrt transformed, MANOVA, F _2, 40_ = 22.5, P < 0.0001). About 28.7 ± 24.4 localities were found for the *S. saevissima Pseudacteon*, 4.2 ± 3.6 localities for all North American taxa and 2.8 ± 3.2 records for the Old World *Pseudacteon.*

**Figure 2.**
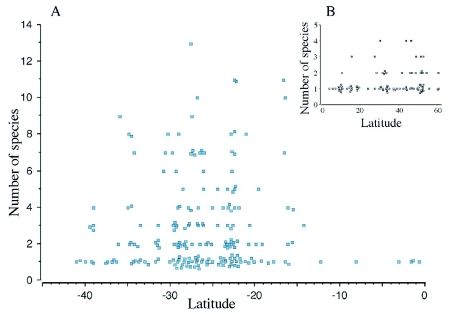
Relationship between latitude and sizes of locality assemblages of *Pseudacteon.* (a) locality assemblages of the *Pseudacteon* associated with the S. *saevissima* complex in South America, (b) locality assemblages in the Northern Hemisphere associated with all hosts.

*Pseudacteon* locality assemblage sizes are shown in Figures 2a and b. Species richness in localities was often substantial, especially for assemblages of those associated with the *S. saevissima* complex (2.8 ± 2.4 species, Figure 2a). Restricting localities to those where only the *S. saevissima* complex *Pseudacteon* was found, single species were found in 115 (44.2%) localities, while two to four species were found in 99 (38.1%) locations and five to 13 species were found in 42 (16.2%) of the 260 recorded localities. Assemblages where the *S. geminata* complex *Pseudacteon* were found were typically less rich with only 21.5% of localities having more than one species. Excluding these localities, that is, including only those for *Pseudacteon* associated with non-*Solenopsis* hosts, only 5.4 % or 9 of 165 localities included two or more species. Taken together, assemblages represented by two or more species were found in 34.6% (161 of 473) localities. Assemblages were larger absolutely in the Southern Hemisphere (Figure 2a) than in the Northern Hemisphere (Figure 2b).

Maps of the native ranges for each species of *Pseudacteon* associated with the *S. saevissima* complex are given in Maps 2–21. An overview of collection sites for these flies is given in Map 1 with an overlay of the native distributional range of the *S. saevissima* complex (Pitts 2002, Cuezzo 1999).

## Discussion

Long distance movements were found to be a recurrent theme in the evolutionary ecology of *Pseudacteon.* For instance, the genus was found in both Eastern and Western Hemispheres. Two species are known only from islands, both in the Malay archipelago, *P. crinifer* Beyer (Bougainville Island, Papua-New Guinea) and *P. javensis* (Java, Indonesia) and eight taxa have both island and continental distributions; *P. antiguensis, P. arcuatus, P. dorymyrmecis, P. grandis*, and *P. simplex*, (West Indies-Americas); *P. brevicauda* (Azores-Continental Europe); and *P. lundbecki* and *P. formicarum* (British Isles-Continental Europe). Generally large geographical ranges were found for many species, including many for which there are only a few collection records (Appendix 1). Given the qualities of small size, short-life span, and non-phoretic tendencies that would limit active *Pseudacteon* flight capacities, these data lend broad support to the Morrison et al. (1999) contention that these phorids can be dispersed widely by wind.

Individual *Pseudacteon* species are host-specific-parasitoids (Porter and Gilbert 2004; Estrada et al. 2006, Weissflog et al. 2008) and their geographical range is limited largely by their host fidelity. For instance, the upper size limit for any species distributional range can only be as large as that of its hosts (but see below). Among *Pseudacteon* associated with the *S. saevissima* complex, this maxima is apparently reached by *P. obtusus.* The geographic distribution of hosts of the flies therefore offers some explanation for variation in the distribution of individual *Pseudacteon* species in addition to that shown for climatic factors (Folgarait et al. 2005). A significant linear relationship was not found when sampling effort between the number of host species of each taxon and its geographical range was accounted for, however, hosts with broad distributions often had *Pseudacteon* with associated extensive ranges (Folgarait et al. 2005). Examples also exist in *Pseudacteon* not associated with *S. Saevissima.* In the New World, the tropical fire ant, *S. geminata*, is found from the southern United States into Brazil and has the largest distributional range of any fire ant. Two *Pseudacteon* species associated with it, *P. antiguensis* and *P. crawfordi* (Appendix 1), have extensive ranges that overlap a substantial portion of this range. If *P. antiguensis* is as host specific as other *Solenopsis* associated taxa, the outline of its range (Appendix 1) suggests that the only host with which it comes into contact with is *S. geminata.* The range of *P. crawfordi*, on the other hand, is also extended through its use of close relatives of *S. geminata* in the western and south-central United States where the tropical fire ant is not found.

The largest geographical ranges, in fact appear to be the result of a species using multiple hosts. This is most striking in South America where the *S. saevissima* complex geographical ranges are smaller than that of the New World range of *S. geminata.* The widest ranging *Pseudacteon* species, *P. obtusus* has been found attacking most of the common *S. saevissima* fire ants including *S. electra* Forel, *S. invicta, S. interrupta* Santschi, *S. macdonaghi* Santschi, *S. quin-quecuspis* Forel, *S. richten* and *S. saevissima* (Smith) as well as *S. gayi* (Spinola). All other *Pseudacteon* hosted by *S. saevissima* complex fire ants with Range Distances greater than the median were also found associated with multiple hosts.

Conversely, one might expect that species with smaller or geographically restricted distributions would be more host limited, either by utilizing species with small ranges and/or by having higher degrees of host-specificity (i.e. Koizumi et al. 1999). This might be the case with *P. conicornis*, for instance, which is known only from *S. saevissima* along the Atlantic coastline of Brazil. Its range appears to be restricted additionally by climate or habitat as *S. saevissima* has a broader distribution in Brazil. In fact, multiple host use occurs even in species with observed minor ranges. *Pseudacteon bulbosus* is found only in the Argentina province of Santiago del Estero but is known to attack two of the *Solenopsis* found in the province, including *S. interrupta* and an unnamed species, *S.* nr. *electra* (Appendix 2) (Brown et al. 2003; Calcaterra et al. 2005; Folgarait et al. 2007a).

We had considered the distribution of the *S. saevissima* complex *Pseudacteon* to be limited to that of the *S. saevissima* host complex but three cases were found where this appears not to be the case. However, the possibility that these three cases could be the result of erroneous location data or disjunct populations of the *S. saevissima* complex cannot be excluded.

*Pseudacteon obtusus* was recently found in central Chile (Calcaterra et al. 2007) attacking *S. gayi*, a taxonomically difficult species that been tentatively placed in the *S. geminata* complex by Pitts (2002). Finding it in Chile, therefore suggested that *P. obtusus* had breached both the high Andes as well as the host division between the *S. geminata* and *S. saevissima* complexes. The host switch to *S. gayi* would likely have been immediate, as well, since no other fire ants are recorded in central Chile to have acted as intermediate hosts (Snelling and Hunt 1975). To resolve this issue, Calcaterra et al. (2007) compared DNA sequences and other molecular characters of *S. gayi, S. geminata* and several *S. saevissima* complex species. Their results suggested an ambiguous membership of *S. gayi* in either complex.

**Map 1.**
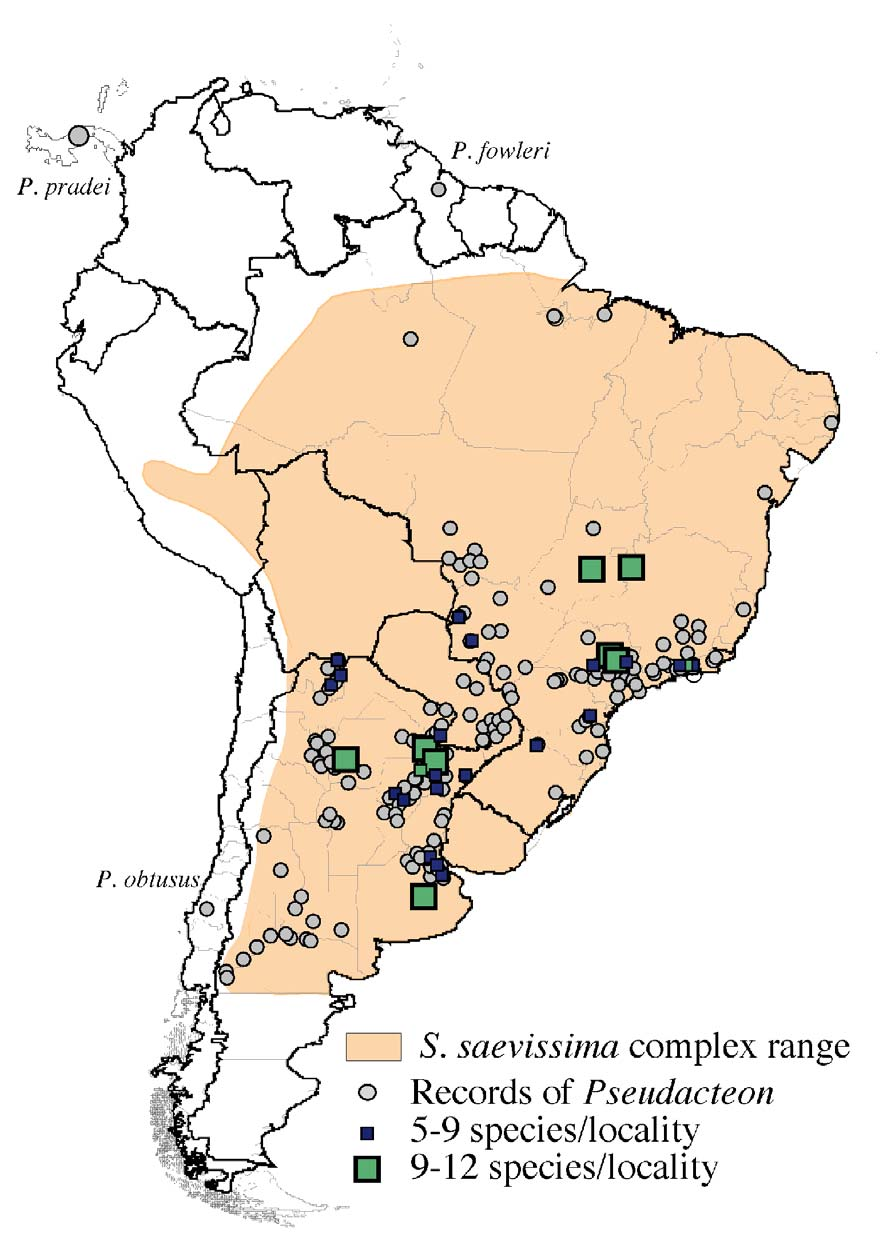
Distributional records for all the *Pseudacteon* species hosted by ants in the *Solenopsis saevissima* complex. Localities with at least 5 species records are depicted with rectangles. These records are superimposed on a map approximating the native range of the host ant complex (after Pitts 2002 and Cuezzo 1999). *Pseudacteon* species names are given for records outside of this range.

One of their analyses, however, demonstrated an overlap in cuticular hydrocarbon components between *S. gayi* and *S. invicta.* Regardless of their phylogenetic importance, it is possible that these or other associated cuticular components may be important for *P. obtusus* recognition of *S. gayi* as a host. An alternative explanation for this host switch is that while Estrada et al. (2006) found a small percentage of Argentine *P. obtusus* that would attack *S. geminata* after being motivated to oviposit on *S. invicta*, this percentage was not null. An absolute absence of a preferred host might lead to a different oviposition behavior for this taxon. A more refined understanding of ovipositional cues used by *Pseudacteon obtusus* or other species might help separate these hypotheses. *Pseudacteon obtusus* has tentatively been established in one site in Texas (Gilbert et al. unpublished observations), and post-establishment host-specificity tests, such as reported for other *Pseudacteon* in Florida (Vasquez et al. 2005; Morrison and Porter 2006), should also be implemented on a continual basis if this or other populations become viable.

Both *P. fowleri* and *P. pradei* were also found outside of the currently recognized boundaries of the *S. saevissima* group (but see Wilson 1952). We initially found the *P. pradei* label location suspicious but the *P. obtusus* and *P. fowleri* records suggest the location might be credible. For both species, only more detailed collecting of both flies and ants will determine whether there has been a host complex shift or whether the boundaries of the *S. saevissima* group might be extended to the north. Host data are not known for either of these records since that for *P. fowleri* was from a malaise trap (Pesquero 2001) and that of *P. pradei* was not noted. Given the tramp species status of *Solenopsis* species it is likely these records may be associated with disjunct populations of species in this complex. An alternative explanation is that the *P. fowleri* from Guyana is a variant of *P. arcuatus*, a similar species found on the *S. geminata* complex ants. Given that intermediate distributional records are lacking for both hosts and flies, these records may represent long-distance dispersal events.

**Maps 2–5.**
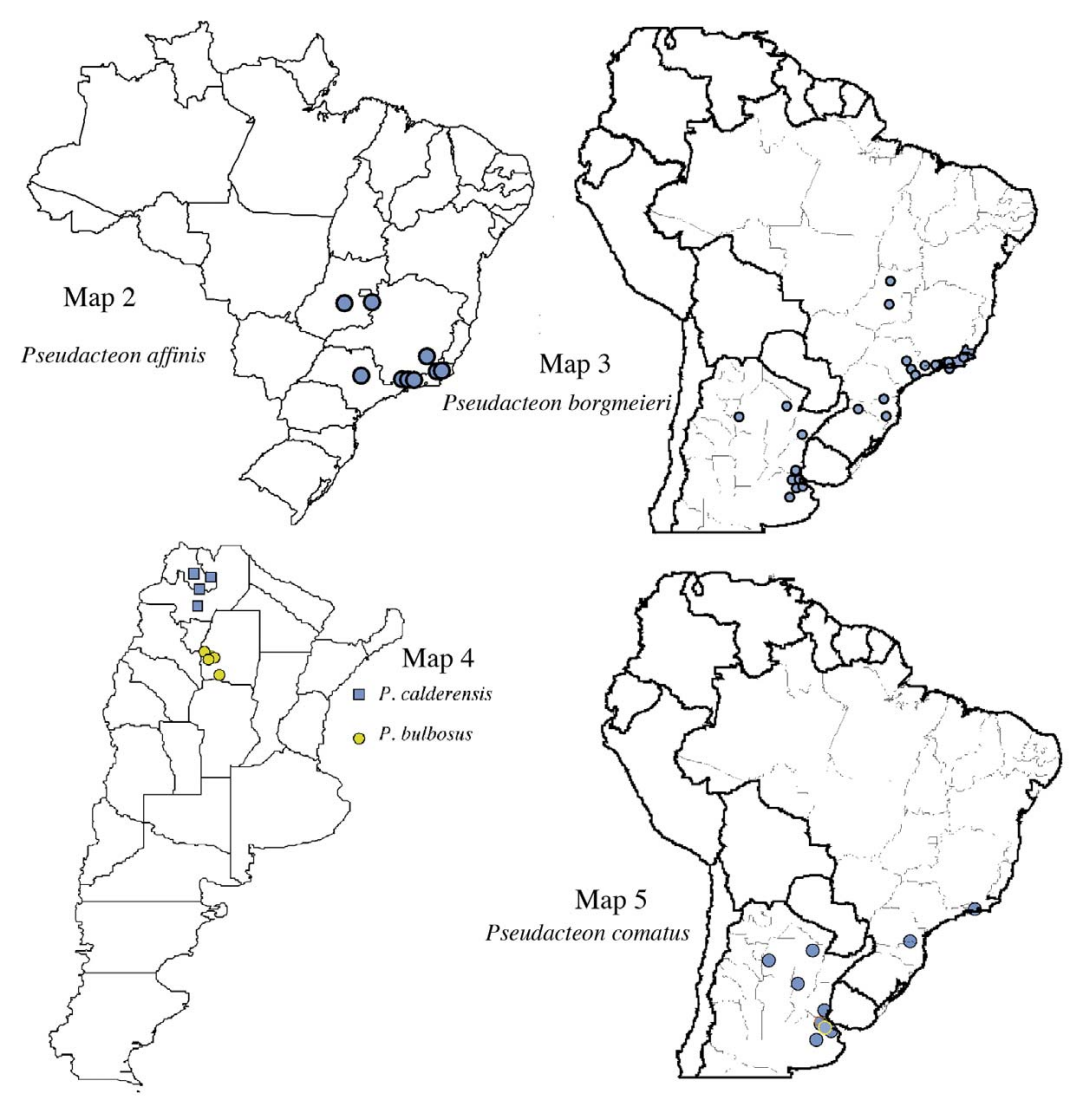
(2) Distribution of *Pseudacteon affinis;* (3) Distribution of *Pseudacteon borgmeieri;* (4) Distribution of *Pseudacteon bulbosus* and *P. calderensis*; (5) Distribution of *Pseudacteon comatus*

We had also expected that the greater organization of effort to find imported fire ant parasitoids might lead to a better understanding of their distributions than for other taxa. This was in fact the case; the resolution of distributions as measured by latitude-longitude points collections was significantly greater for the South American *S. saevissima Pseudacteon* than for other New World or Old World taxa. Although sampling bias with respect to sources certainly contributes to this finding, it is noteworthy that Range Distances did not differ significantly across host complexes. Sample sizes/taxa were nearly 6 times higher for the *S. saevissima* than the *S. geminata* complex *Pseudacteon.* Collections for the *S. saevissima* complex *Pseudacteon*, however, have been much more intense in smaller areas than that for other taxa (i.e. Folgarait et al. 2007a, b; Folgarait et al. 2005; Borgmeier and Prado 1970; Williams and Whitcomb 1974) resulting in finer resolution of locally rare taxa that might have been missed with more casual collecting. As an example, the Range Distance observed for *P. comatus* was slightly higher than the median for the genus (2105 vs. 2098 km) though this taxon was often extremely rare; it was collected in just 0.03% of samples in one very intensive study (Folgarait et al. 2007a) and its numbers ranked last or nearly last of all *Pseudacteon* collected in other extensive surveys (Williams 1980; Folgarait et al. 2003; Calcaterra et al. 2005). Other species such as *P. nocens* or *P. curvatus* which are very abundant and widespread in northern Argentina are rare in parts of Brazil and like *P. comatus*, may not have been collected in such areas without systematic efforts.

**Maps 6–9.**
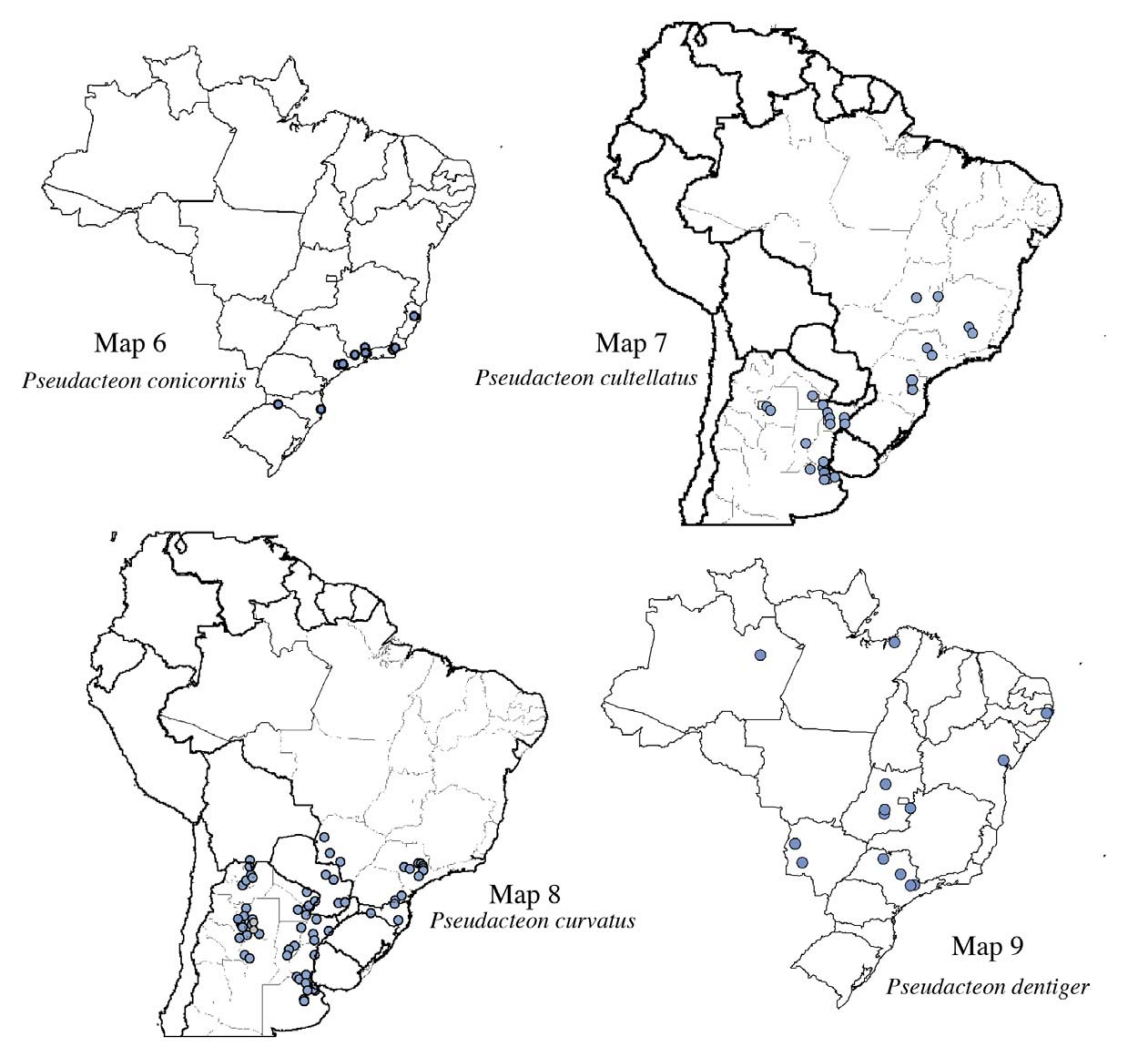
(6) Distribution of Pseudacteon conicornis; (7) Distribution of *Pseudacteon cultellatus*; (8) Distribution of *Pseudacteon curvatus*; (9) Distribution of *Pseudacteon dentiger*

The examples given above indicate that the relationships between measures of local abundance and range breadth for many of the *Pseudacteon* associated with the *S. saevissima* complex are best seen on a case-by-case basis. Almost all of the widespread taxa (having Range Distances greater than the Median) such as *P. obtusus, P. pradei, P. litoralis* and *P. tricuspis* were collected in typically higher numbers than other taxa, but species such as *P. wasmanni, P. curvatus*, and *P. nocens*, though also quite common in much of their range, had Range Distances less than the median. Some of the lesser common taxa such as *P. bulbosus*, *P. conicomis* had narrow ranges while the ranges of *P. fowleri* and *P. comatus* seem to be over sampled based on numbers collected. One might have expected that the Range Distances for trail specialists (Orr et al. 1999) such as *S. solenopsidis* might have been noticeably under sampled because they are typically found in much lower numbers than mound specialists, such as *P. curvatus* and *P. tricuspis* but this was not observed; again, this finding is probably due to the systematic efforts and collecting techniques designed to find *Pseudacteon* associated with *S. saevissima* complex hosts.

**Maps 10–13.**
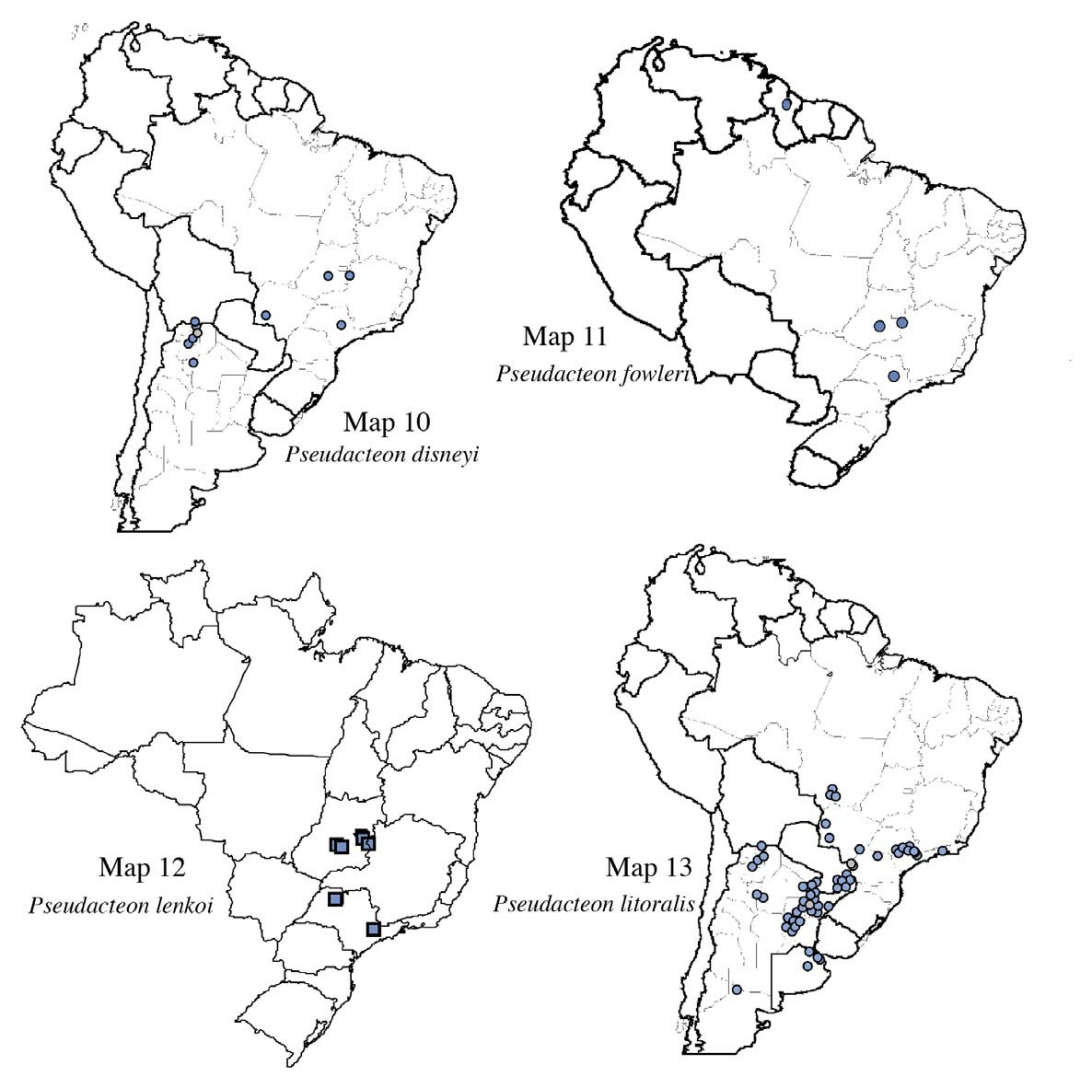
(10) Distribution of *Pseudacteon disneyi*; (II) Distribution *of Pseudacteon fowleri*; (12) Distribution of *Pseudacteon lenkoi*; (13) Distribution of *Pseudacteon litoralis*

Multiple species were found in the majority of the locality assemblages hosted by *S. saevissima* complex ants (Figure 2a). This finding is a technical overestimate in that localities with no observed phorids were not included in the database. In other instances, sites may have been pooled because of the non-specificity of the locality record or because records were collected over a number of years. Still, it is striking that large assemblages of these flies are relatively commonplace. Folgarait et al. (2007a), for instance found 12 species at one site comprising approximately a square decameter and they and Calcaterra et al. (2005) reported finding up to 9 species in a single day in a site. While we found that assemblages on other hosts and areas were smaller, it should be noted that the species pool for these assemblages were also substantially smaller. We are also very likely to have underestimated species richness for these assemblages due to less collection effort. The estimate, for instance, of less than one-quarter of localities being represented by two or more *Pseudacteon* associated with *S. geminata* complex ants is difficult to accept based on collection records for localities with multiple sampling periods (Ed Lebrun 2007, personal communication; RJWP, unpublished observation).

**Maps 14–17.**
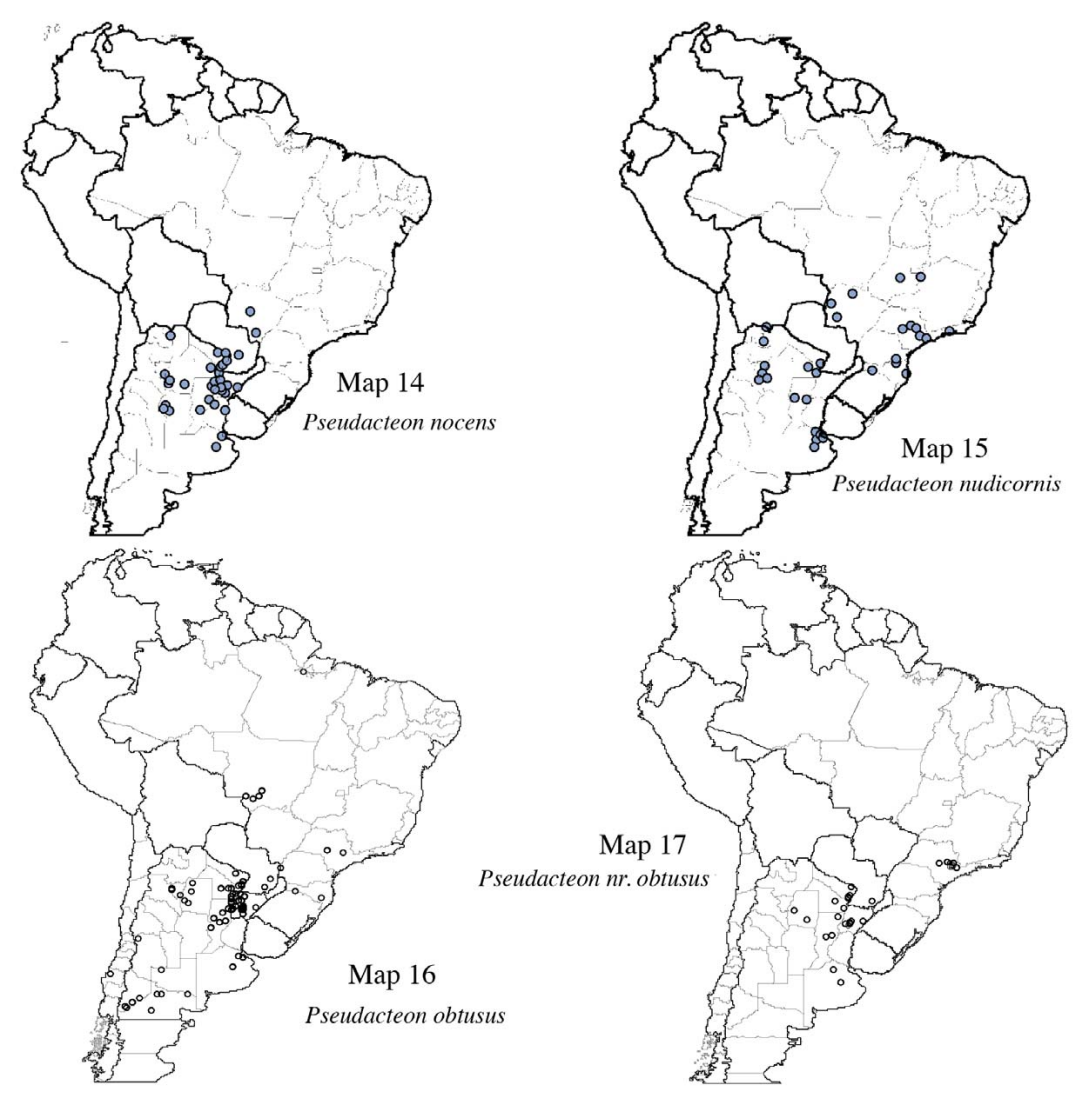
(14) Distribution of *Pseudacteon nocens*: (15) Distribution of *Pseudacteon nudicornis*; (16) Distribution of *Pseudacteon obtusus*; (17) Distribution of *Pseudacteon* nr. *Obtusus*

The generally wide distributions for many of the taxa suggests one reason why there are many places where multiple-species communities are found (Map 1, Figure 2), that is, if their distributions were more narrow, there would be fewer overlapping ranges. Explanations given for the frequency of multiple *Pseudacteon* species complexes found have largely centered on degrees of niche partitioning (Orr et al. 1997, Morrison 2000). With increased intensive sampling, it is likely that many of the localities where only one species is documented will result in larger observed communities, especially for the *S. saevissima* complex *Pseudacteon.* The observed large overlap in distributions at both micro and macro scales will make finding differences in the fundamental and realized niches of taxa expected by niche partitioning more difficult, but see Folgarait et al. (2007 a, b) who did find differences. Ecological studies with artificially generated *Pseudacteon* communities of various sizes in the United States resulting from classical biological control efforts will therefore be of theoretical interest in comparison to studies done in the native range of the *S. saevissima* host group.

The distributional data presented here includes records that are based on identifications by authorities, as well as through the use of diagnostic keys. Identification of *Pseudacteon* is most often made using the females, since ovipositor shape is typically diagnostic (Porter and Pequeno 2001). Cryptic taxa were recently, however in populations of *P. obtusus* and may be present in other populations (i.e. *P. tricuspis*; Porter and Pequeno 2001). Kronfurst et al. (2007) found three evolutionary lineages of *P. obtusus* including two large and one small morpho-types. The smaller morpho-type, which was designated as *P.* nr. *obtusus*, is sufficiently different from the other morphotypes to allow mapping its distribution where specimens or specific records are available (Map 17). We cannot separate the two larger morpho-types at present, however, There is considerable micro and macro geographical overlap between the small and larger morpho-types and older records for *P. obtusus* might represent one and or any of these cryptic taxa. Potential for confusion over distributional ranges of these cryptic taxa is illustrated by records from São Paulo, where *P. obtusus* has been very intensively collected for classical biological control studies. Examination of available specimens by SDP showed that all records, with the exception of one for *P. obtusus* were actually for the smaller morph, *P.* nr. *obtusus.* Problems with taxonomic identity may also be present in the distributional range of *P. tricuspis* where one form occurs on *S. saevissima* *and S. invicta* ants and another form occurs on *S. richteri* ants (Porter and Pesquero 2001). It is very likely that an exacting phylogenetic analysis of the genus will alter and potentially reduce the distributional ranges of some of the taxa, including that of *P. obtusus* from that presented here.

**Maps 18–21.**
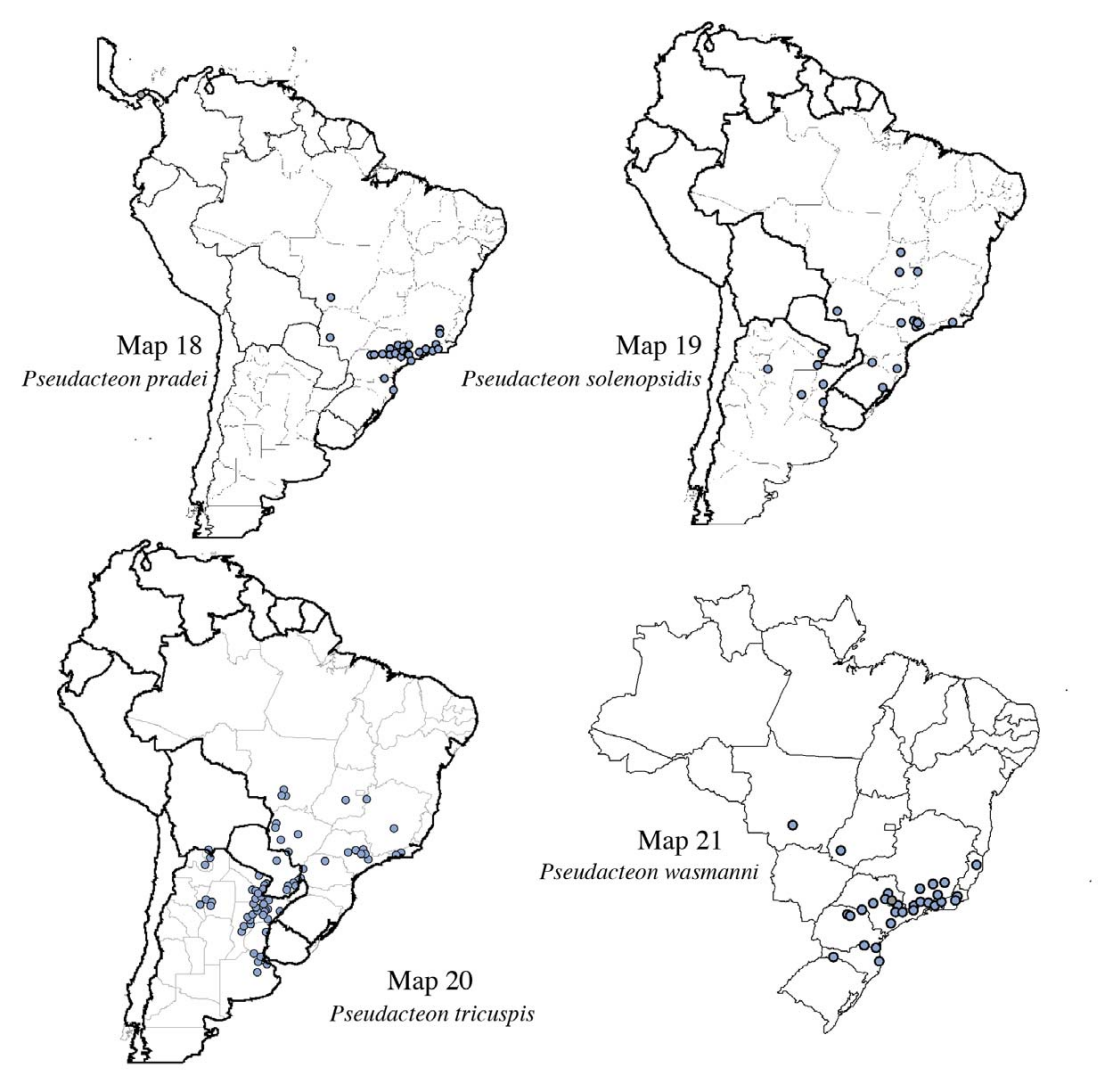
(18) Distribution of *Pseudacteon pradei*; (19) Distribution of *Pseudacteon solenopsidis*; (20) Distribution of *Pseudacteon tricuspis*; (21) Distribution of *Pseudacteon wasmanni.*

One of the agendas in mapping species distributions is to inform collectors as to areas not yet or poorly explored. While the *S. saevissima* associated *Pseudacteon* are found broadly over much of their hosts' range, there are many substantial gaps in our knowledge of their ranges (Map 1). First, the vast and rugged distances in the interior of Brazil and other countries of South America is one persistent and obvious factor underlying why *Pseudacteon* records are completely absent or at best spotty in most areas north of 20 °S (Map 1, Figure 2). Second, there has been very little exploration south of the northern strip of Buenos Aires province (but see Calcaterra et al. 2007) despite the widespread occurrence of hosts in this region. This deficiency can be seen as well in Figure 1 by the extended line of taxa symbols at 35 °S. Understanding the southern distributional limits of *Pseudacteon* in South America could help make predictions about the possible limits of northern expansion of introduced species used in classical biological control of the imported fire ants in North America. Third, relatively little is known about the western distribution of these flies, particularly along the eastern border of the Andes. Much of this area is relatively arid and although cold, could have populations that might be serve as sources of flies for classical control in areas along the margins of the imported fire ant newly accessed distributions in Texas (Gilbert and Patrock 2002). The observed ranges of several taxa across extremes of climate from the lowland tropics of Brazil to the cold Monte of Argentina and higher elevations along the foothills of the Andes (Calcaterra et al. 2005, Calcaterra et al. 2007), suggests there are populations of flies that might fit a wide range of climatic conditions for classical biological control efforts in most areas of the imported fire ants invasive range.

Towards the east, there are no records for Uruguay. This area is largely occupied by the black imported fire ant and its exploration might prove useful for clarifying the southern limits and host usage patterns of some of the ‘endemic’ species of Barzil. Finally, within the ‘collecting space’ better sampled (Map 1), there are significant gaps that represent unsampled phytogeographical areas or distributional transitions among hosts. The most obvious and compelling areas for examining host shifts would be the transitional boundary areas between *S. invicta* and *S. dchted*, as well as those of *S. invicta* and the nominal *S. saevissima.* Exploration of these areas could disclose information pertinent to understanding niche or host shifts for those species present. For the *Pseudacteon* not associated with *S. saevissima* complex, of course, there are relatively few locations described and these have been less collected. Given that the *Pseudacteon* is found with ants on all the non-polar continents our meager knowledge indicates there is much to be described.

*Pseudacteon* are tiny, fast-moving flies that are not frequently seen or captured away from their hosts using passive devices such as pan or malaise traps (Orr et al. 2003; Carles-Tolrá 2006). Their presence near ants is often very patchy, both spatially and temporally (Folgarait et al. 2007a) which may be an additional reason why they may be overlooked. Techniques used in the study of *Pseudacteon* associated with *Solenopsis* fire ants may help in documenting the distribution of other species, as well as uncovering other *Pseudacteon* host interactions. Using chemical cues, including the presentation of dead ants (Smith and Gilbert 2003), or aroused live hosts (Orr et al. 2003; Barr and Calixto 2005), and especially the use of sticky traps (LeBrun et al. *in press*; Puckett et al. 2007), have proven useful in documenting the presence of these flies at low densities and could be manipulated to search for taxa associated with other ants. While many of these flies could easily be seen as rare and localized based on our current understanding, these components of rarity are related to collecting effort and the quality of visualizing methods (Carles-Tolrá 2006). The fascinating biology of the interactions among these flies and their hosts warrant additional work in both effort and method.

## Notes

### Editor's Note

Paper copies of this article will be deposited in the following libraries. Senckenberg Library, Frankfurt Germany; National Museum of Natural History, Paris, France; Field Museum of Natural History, Chicago, Illinois USA; the University of Wisconsin, Madison, USA; the University of Arizona, Tucson, Arizona USA; Smithsonian Institution Libraries, Washington D.C. USA; The Linnean Society, London, England.

## Figures and Tables

**Appendix 1.**
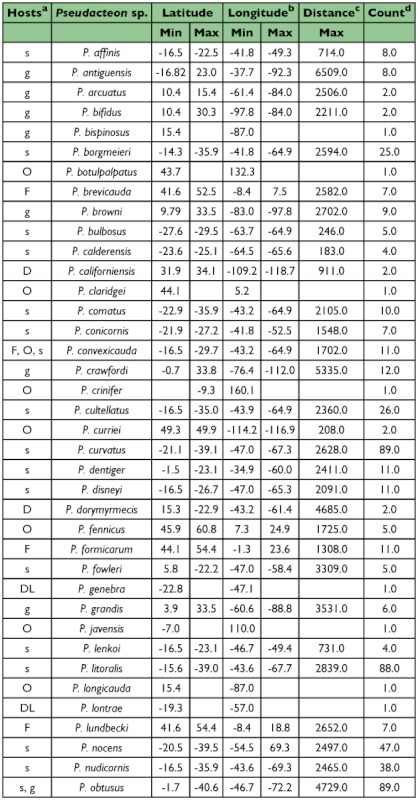
Geographical ranges (latitudinal and longitudinal mimimal and maximal degrees) and Range Distance Maxima (km) for the different species of Pseudacteon.

**Appendix 2.**
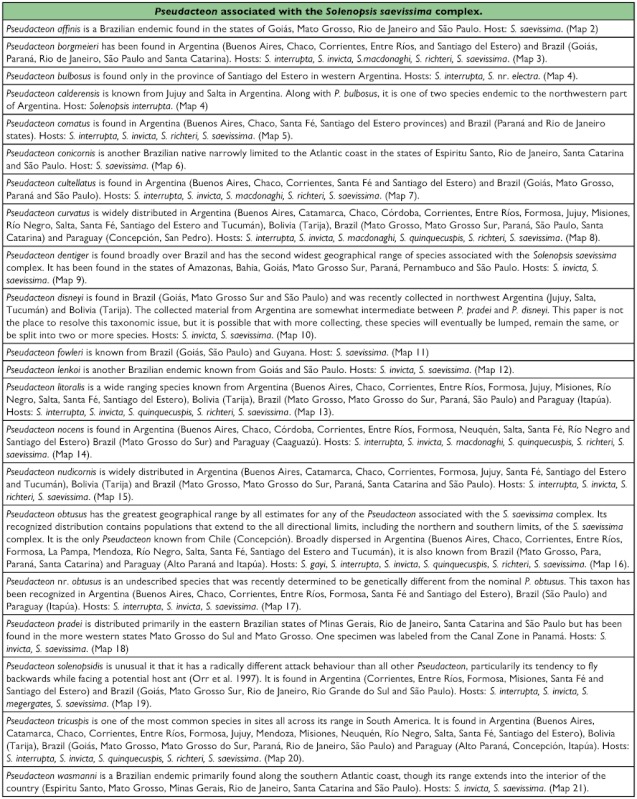
A listing of the Pseudacteon associated with Solenopsis saevissima fire ants with the countries and secondary order administrative areas where each has been identifed, as well as host information. Host data provided is based only on field associations since the majority of species have not been reared.

**Appendix 3.**
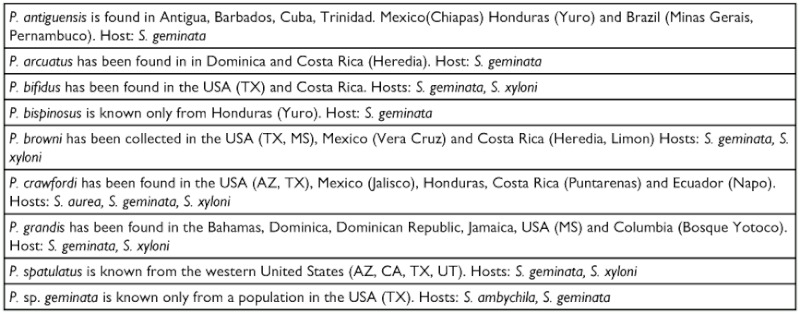
A listing of the Pseudacteon associated with Solenopsis geminata fire ants with host countries and secondary order administrative areas where each has been identifed, as well as information. Host data provided is based only on field associations since the majority of species have not been reared.
